# Understanding the Impact of Face Masks on the Processing of Facial Identity, Emotion, Age, and Gender

**DOI:** 10.3389/fpsyg.2021.743793

**Published:** 2021-11-03

**Authors:** Daniel Fitousi, Noa Rotschild, Chen Pnini, Omer Azizi

**Affiliations:** Department of Psychology, Ariel University, Ariel, Israel

**Keywords:** COVID-19, masks, face perception, social perception masks and face perception, social perception

## Abstract

The COVID-19 pandemic has introduced new challenges for governments and individuals. Unprecedented efforts at reducing virus transmission launched a novel arena for human face recognition in which faces are partially occluded with masks. Previous studies have shown that masks decrease accuracy of face identity and emotion recognition. The current study focuses on the impact of masks on the speed of processing of these and other important social dimensions. Here we provide a systematic assessment of the impact of COVID-19 masks on facial identity, emotion, gender, and age. Four experiments (*N* = 116) were conducted in which participants categorized faces on a predefined dimension (e.g., emotion). Both speed and accuracy were measured. The results revealed that masks hindered the perception of virtually all tested facial dimensions (i.e., emotion, gender, age, and identity), interfering with normal speed and accuracy of categorization. We also found that the unwarranted effects of masks were not due to holistic processes, because the Face Inversion Effect (FIE) was generally not larger with unmasked compared with masked faces. Moreover, we found that the impact of masks is not automatic and that under some contexts observers can control at least part of their detrimental effects.

## 1. Introduction

Faces are among the most important stimuli in our environment. They convey information regarding many primary attributes such as: identity, emotion, gender, and age (Bruce and Young, 1986). Normal processing of faces is essential for social perception and interaction. However, recently the coronavirus disease 2019 (COVID-19) pandemic has exerted dramatic influences on the way we process faces. Unprecedented efforts at reducing the devastating effects of the pandemic have culminated in practices such as social distancing and mask-wearing. In many countries over the glob governments require their citizens to wear masks in public in order to reduce virus transmission. This new constraint has opened a whole new arena for social perception of partly occluded faces. Given the considerable importance of intact face processing in social interaction, it is important to understand how face masks affect face recognition. A great deal of studies have been dedicated recently to understanding the impact of face masks on various aspects of social interaction and cognition (Carbon, 2020; Carragher and Hancock, 2020; Freud et al., 2020; Green et al., 2020; Spitzer, 2020; Carbon and Serrano, 2021; Gori et al., 2021; Grundmann et al., 2021; Kastendieck et al., 2021; Marler and Ditton, 2021; Molnar-Szakacs et al., 2021; Noyes et al., 2021; Stajduhar et al., 2021). Many of these studies have demonstrated the detrimental effects of masks on the recognition of facial emotion and identity. These studies have harnessed non-speeded tasks and measures. The current study expands the scope of investigation to speeded measures and to additional primary facial dimensions such as gender and age. The study offers novel insights into the impact of face-masks on face perception and on the underlying mechanisms. We show that surgical masks impair both speed or/and accuracy of face categorization. We also find that these disruptions are largely not due to impaired holistic processing.

The effects of face masks on human interaction and cognition might be far-reaching than one may imagine. Faces are important sources for expression and extraction of valuable information about ourselves and others. Therefore, acting in a world in which faces are partially occluded poses serious challenges to normal human performance in many scenarios. These include, but not restricted to, schooling (Nobrega et al., 2020), caregiver-patient relations (Marler and Ditton, 2021), and people with hearing loss who benefit from lip-reading (Chodosh et al., 2020). All of these scenarios are significantly impeded when actors are wearing masks. To overcome, at least partially, the devastating consequence of masks, and to be able to offer remedies, we need to first understand how face masks interfere with basic aspects of face recognition. What primary dimensions are most vulnerable to masks? How speed and/or accuracy are affected by masks? And are there certain contexts under which people can circumvent the detrimental effects of masks?

## 2. How Masks Affect Face Recognition?

The vast literature on face masks addresses a broad scope of issues. Here we focus on studies that tested for the effects of masks on basic process of face recognition, in particular the perception of identity and emotion. Research dating from the pre COVID-19 era with masked and occluded faces has revealed a reduction in recognition of facial identity (Dhamecha et al., 2014). In one study (Stephan and Caine, 2007) recognition accuracy of familiar and unfamiliar faces declined when different features of the face (e.g., mouth) were removed. In another study (Fitousi and Wenger, 2013), recognition of identity was hampered (in both accuracy and speed) when sunglasses were added. Similarly, categorization of facial expression was disrupted when a scarf covered the mouth area (see also, Kret and De Gelder, 2012; Noyes et al., 2021). In one of the first COVID-19 studies by Carragher and Hancock (2020) participants judged whether two simultaneously presented faces showed the same personal identity or two different identities. Surgical masks had a large detrimental effect on face matching performance irrespective of whether one or two of the faces were masked. The impairment was similar in size for familiar and unfamiliar faces. Other studies have shown that faces reduce emotion recognition accuracy (Carbon, 2020; Grundmann et al., 2021), or the intensity of such facial emotions as happiness and anger (Calbi et al., 2021). In one important study, Carbon (2020) presented faces with six different emotional expressions in either a fully visible condition or in a partly covered (with masks) condition. He found lower accuracy and confidence levels in the masked faces condition. In addition, observers misinterpreted disgusted faces as being angry, while other emotions (e.g., happy, sad, and angry) were assessed as neutral. All of these studies suggest that face masks interfere with basic mechanisms of face recognition to some degree. But what are those mechanisms that are responsible for the impairment?

Previous studies (Carragher and Hancock, 2020; Freud et al., 2020) have largely focused on the question of how masks affect recognition accuracy of facial identity or facial emotion (Carbon, 2020; Grundmann et al., 2021). While most of these efforts pointed to the dramatic effects of masks on these aspects, many practical and theoretical issues have remained unanswered: (a) the degree to which masks affect speed of processing of faces, (b) the influence of masks on other primary facial dimensions such as age, and gender which play a central role in social cognition (Darwin, 1872; Freeman et al., 2012; Cloutier et al., 2014), (c) the mechanisms that may be responsible for these effects, and finally (d) the role of strategies in producing these effects. All of these bear tremendous import for more advanced processes that depend on correct and efficient initial person construal processes (Freeman and Ambady, 2011; Fitousi, 2020b), such as impression formation and stereotyping (Oosterhof and Todorov, 2008).

## 3. Do Faces Masks Exert Selective Influence?

The recent literature on face masks may lead us to think that face masks universally distort the recognition of all types of facial dimensions (e.g., gender, emotion), under all conditions, and with respect to all types of performance measures. However, it is imminently possible that the influence of masks is selective, with some facial attributes being immune to such influences. It might be the case that certain facial dimensions can be efficiently extracted from face areas that are not occluded by the mask. Carbon (2020) has shown that recognition of fear (in contrast to other emotions) was not hampered by masks, probably because fear is decoded from the top part of the face. Similarly, Kastendieck et al. (2021) have found that masks did not interfere with recognition of happiness and sadness in dynamic emotion expression.

So how can one predict what face attributes are going to be distorted by masks? Face researchers have developed methods to “reverse-engineer” the mind, with the goal of uncovering the mappings between psychological dimensions (e.g., emotion) and the corresponding facial features/areas (e.g., mouth) that are necessary for their decoding (Gosselin and Schyns, 2001; Abudarham and Yovel, 2016; Abudarham et al., 2019). The “Bubbles” technique (Gosselin and Schyns, 2001), for example, allows researchers identifying the specific visual information that is relevant for specific categorizations. Using this technique (Blais et al., 2012) have shown that the mouth area (and not necessarily the eyes) is the window to emotions. Measuring categorization performance with faces wearing masks can serve as a “reverse-engineering” technique by its own right. It can inform us on the importance of the mouth area in the categorization of various face dimensions.

A related issue that deserves a comment concerns the deployment of strategies. It is also plausible that observers have developed specific strategies during the COVID-19 period to circumvent the obstacles posed by masks. In natural everyday circumstances we can still interact with other people, as well as perceive their identity, gender, age, emotion and other social aspects, in spite of the debilitating conditions of masks. This is also true with respect to other sub-optimal conditions under which we perceive faces such as: poor lighting, low acuity, deformation in shape, in which we can still recognize faces quite efficiently. People have learned to cope with such debilitating conditions by harnessing unique processing strategies. For example, people can take advantage of additional contextual cues from voice (Golan and Baron-Cohen, 2006; Knoblauch et al., 2021), gate (Cutting and Kozlowski, 1977), body gestures (Aviezer et al., 2012), and the social context of a situation (Mondloch, 2012) to make correct inferences on whether a face belongs to a given category. Moreover, not all aspects of performance (e.g., speed) should suffer to the same extent. For example it might be the case that people trade off their accuracy for speed, or alternatively, compromise their speed to achieve high levels of accuracy. Research programs should allow us to better understand the relations between speed and accuracy and the circumstances under which such strategies may arise.

## 4. Potential Mechanisms for the Effects of Masks

A plausible candidate for the detrimental effects of masks on face recognition is the disruption of a psychological mechanism known as *holistic processing* (Young et al., 1987; Farah et al., 1998). According to this idea, faces are perceived as wholes, and the constituting parts or features are secondary to the global Gestalt (but see, Fitousi, 2015, 2016, 2019, 2020a). Disruption of holistic processing mechanisms through occlusion of face parts may lead to an impairment in face recognition. Researchers (Maurer et al., 2002) have distinguished between three types of holistic representations: (a) first-order relations (e.g., two eyes above a nose), (b) Gestalt global form (e.g., the entire face), and (c) second-order relations between features (e.g., distance between the eyes). Face masks cover areas of the mouth and nose, and therefore might hamper any (or all) of these types of representations. In a large-scale online study, Freud et al. (2020) assessed face processing for masked and unmasked faces using an adapted version of the Cambridge Face Memory Test. As predicted, they found considerable decrease in performance in this test with masked faces. Interestingly, the face inversion effect (FIE, Yin, 1969)—impaired performance with inverted faces—was smaller with masked compared to unmasked faces. The FIE (Prete et al., 2015a,b) is often considered as a marker of holistic processing (Farah et al., 1995), and the authors interpreted the effect of masks on the FIE as evidence that masks disrupt holistic (first-order and Gestalt) processing of faces. This argument is supported by other studies showing that holistic processing is important for normal face perception abilities, and that it is disproportionally contributed by the lower (mouth region) part of the face compared with the upper (eye region) part (Tanaka et al., 2014). However, it should be noted that some researchers question the view that the FIE is a marker of holistic processing (Sekuler et al., 2004).

Another source of evidence for the role of the lower part of the face in holistic face recognition comes from the *composite face effect* (CFE, Young et al., 1987). In this paradigm, participants are presented with a face that is composed from top- and bottom- halves of two different identities. This composite face creates the illusion of a newly, never-seen-before face. There are several versions of paradigm (Fitousi, 2015), but the most used task is often to judge the top part of the face and ignore its bottom part. The typical result shows that when the two face halves are aligned, participants cannot ignore the identity of the bottom part, this effect is reduced or abolished when two face halves are misaligned. The CFE has been often taken as evidence for holistic processing of faces. The CFE illusion is typically observed in tasks that require discrimination between two identities, but it has been also documented in tasks that call for emotion (Calder et al., 2000), gender (Chen et al., 2018) and age (Gray et al., 2020) categorization. In these versions, for example, people tend to perceive the top half of the face as less masculine if the lower part is more feminine. This illusion gives currency to the hypothesis that the lower part of the face is essential for the categorization of identity, emotion, gender, and age. However it should be noted that not all researchers hold to the view that faces are processed holistically (Tversky and Krantz, 1969; Massaro and Friedman, 1990; Donnelly et al., 2012; Fitousi, 2015, 2019; Cheng et al., 2018).

## 5. Predictions

The current study addresses various theoretical and practical issues. First, we were interested in the impact of face masks on the speed and accuracy of categorization of primary facial dimensions. Second, we wanted to uncover the mechanisms governing potential disruptions to normal processing. Third, we tested for the presence of unique processing strategies. All of these issues are addressed here in a systematic and comprehensive fashion. In four speeded classification experiments (*N* = 116), we measured both RTs and accuracy with masked and unmasked faces. The face stimuli consisted of realistic images created in our lab by photographing volunteers with and without masks. Each experiment focused on one facial dimension (e.g., gender) out of the four tested (i.e., identity, emotion, age, and gender). We also tested for the FIE, with face images appearing either upright or inverted. In addition, we probed the existence of processing strategies using blocked and mixed designs.

Our hypotheses were as follows: (a) given the importance of the lower part of the face in many aspects of face categorization, we hypothesized that face masks will disrupt both accuracy and speed in all types of categorizations (identity, emotion, gender, and age) with upright-faces. If indeed, the source of this effect is disruption of holistic processing (Freud et al., 2020) then one should expect that (b) the effect of masks to be reduced or abolished for inverted faces. In contrast, if holistic processing does not play a role in face recognition (Fitousi, 2015), then inversion should not interact with the effect of face masks. In addition, we predicted that (c) if observers are capable of developing strategies for coping with the unwarranted effects of face masks, they should be more likely to do so in blocked- compared to mixed designs. This is because the regularity of the faces in this type of blocks should allow participants to exert top-down control and mitigate the unwarranted effects of masks.

## 6. Methods

### 6.1. Participants

A total of 116 participants took part in Experiments 1–4. Thirty participants were tested in Experiment 1 (mean age = 23.38, *sd* = 2.11, *F* = 22, *M* = 8), 30 In Experiment 2 (mean age = 22.4, *sd* = 2.47, *F* = 25, and *M* = 5), 24 in Experiment 3 (mean Age = 22.87, *sd* = 2.20, *F* = 17, and *M* = 7), and 32 in Experiment 4 (mean Age = 23.34, *sd* = 1.69, *F* = 22, and *M* = 10). They were recruited from Ariel University pool of participants and compensated with a course credit. All observers had normal or corrected-to-normal vision. The study was approved by Ariel University Ethical Committee (AU-SOC-DF-20210411).

### 6.2. Stimuli and Apparatus

The faces in the four experiments were realistic gray-scale images created in our lab. This is important because the great majority of previous studies have not taken pictures with real people wearing masks, but have artificially patched an image of a generic mask on images of unmasked faces (Carragher and Hancock, 2020; Freud et al., 2020). This practice is not ecologically valid because real masks can convey valuable structural information via curvature, depth, light-shading, and other types of cues. This information is lost when the masks are added with a photo editing software to an originally unmasked face.

**Figure 1:**
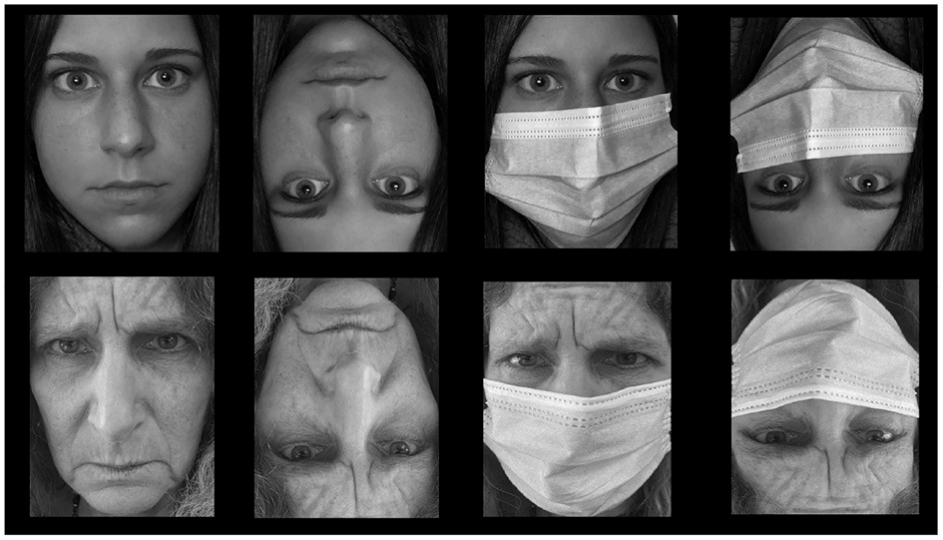
Examples of face images used in the four experiments. The top row presents an example for a young female with neutral emotion. The bottom row gives and example for an old female with angry emotional expression.

Our images conveyed front-views of real people of Caucasian ethnicity, who were photographed with and without a surgical mask covering the lower part of their face including the mouth and the nose (see Figure 1). The faces in our study varied on four primary dimensions: (a) identity, (b) emotion, (c) age and (d) gender. There is a broad consensus among face researchers that these dimensions are the most important for social interaction (Freeman and Ambady, 2011; Fitousi, 2020b). To create the face stimuli, we recruited 16 volunteers from Ariel community area who agreed to take part in a photo-shooting session. All volunteers provided written consents in which they gave their permission to use the pictures in experiments and journal publications. The 16 volunteers consisted of four young females, four young males, four old females, and four old males. The young actors were in their twenties whereas the old actors were in their sixties or seventies. The same photographer took four front-view photos of each volunteer: (a) neutral expression without a face mask, (b) neutral expression with a face mask, (c) angry expression without a face mask, and (d) angry expression with a face mask. All images were taken as color pictures under identical position and light conditions. The color images were then converted into gray-scale images and standardized with a commercial photo editing software. All the faces appeared within a standard rectangle 9 × 7 cm. In total we created 64 images (16 × 4). We then elected the best images of 8 identities (four males and four females) out of the 16 identities. This resulted in 32 images. The faces varied on four primary facial dimensions: age, gender, emotion, and identity. They could be clustered according to a factorial design with 8 levels of identity × 2 levels of gender (male and female) × 2 levels of age (young, old) x 2 levels of emotion (neutral, angry) × 2 levels of mask (no mask, mask) × 2 level of inversion (upright and inverted). This allowed us a tight control over the stimuli set.

### 6.3. Procedure and Design

The exact same set of faces was deployed in all four experiments. The experiments were also identical with respect to the procedure and design. They only differed with respect to the target facial dimension to which the participants were asked to pay attention. That is, on each experiment, participants categorized the faces on a single predefined facial dimension (e.g., gender) out of the four dimensions the faces varied on (i.,e., age, gender, emotion, identity). The task was a speeded two-choice categorization. Participants pressed on one of two keys to indicate their decision. A different group of participants was recruited for each experiment. It should be noted that none of the 116 participants was familiar with the identities of the faces in the experiments. This was confirmed before the experiment took place. In this sense, the faces were unfamiliar.

In Experiment 1, participants categorized the faces according to the face's identity. We grouped the 8 identities in the stimuli set into two artificial social groups (A vs. B). We kept the values of the other three facial dimensions (age, gender, emotion) equally distributed across these two groups. Thus, each group consisted of the same number of men/women, young/old, and neutral/angry faces. On each trial, participants were presented with a face from one of the two groups and had to decide, while timed, whether that face belonged to group A or B. To reduce the potential influence of memory load, we presented small images of the faces of each group on the bottom of the screen on each trial. Participants could therefore use these images to memorize the correct grouping. Because the faces were arbitrarily assigned to each group, the groups had no unique characterizations, and were described to the participants as simply group A and group B. It should be noted that the “identity” of faces in this task is more of a demographic identity rather than a psychological identity. The task does not require familiarity with the identities or deep semantic analysis. In this sense, the task is comparable to the traditional 'same-different' task in which participants judge whether two simultaneously presented face photographs show the same person or two different people (Carragher and Hancock, 2020).

In Experiment 2, participants classified the faces according to the face's emotion (neutral vs. angry). In Experiment 3, the target dimension was age (young vs. old). In Experiment 4, participant categorized the faces according to gender (man vs. woman).

Each experiment consisted of 6 blocks: Block (1)—upright faces with half of the faces wearing face masks and half of the faces without masks, Block (2)—upright faces with all faces wearing masks, Block (3)—upright faces with no masks, Block (4)—inverted faces with all faces wearing masks, Block (5)—inverted faces with no masks, and Block (6)—inverted faces with half of the faces wearing face masks and half of the faces not wearing masks. In combination, the six experimental blocks created two nested experimental designs. Blocks (2)–(5) formulated a blocked design in which the factors of Mask (mask, no mask) and Inversion (upright, inverted) were manipulated across blocks. Blocks (1) and (6) created a mixed design, with the factor of Mask (mask, no mask) being manipulated within a block, while the factor of Inversion (upright, inverted) being manipulated across blocks. Both designs were administrated within-subject with repeated measures. The order of blocks was random. We purposely elected to use these two types of designs to test whether participants will develop specific strategies to cope with the potentially delimiting influence of face masks. Such strategies (if exist), should most likely surface in the blocked design, but not in the mixed design. We hypothesized that the results in the two designs should be identical if participants do not develop unique strategies.

Block (1), which was part of the mixed design, consisted of 32 trials with equal number of personal identities, young and old, male and female, neutral and angry faces (2 identities × 2 genders × 2 age × 2 emotions x 2 mask level). All the faces were presented in an upright position. Block (6) which was also part of the mixed design, consisted of the same 32 faces from Block (1), but now in an inverted position. Blocks (2)–(5) were part of the blocked design. Each of them included 16 trials, which incorporated half of the trials from Block (1). The frequency of dimensional levels was balanced also in these blocks, with equal number of identities, gender, and emotion per block. Block (2) consisted of only masked faces in an upright position. Block (3) presented only faces with no masks in an upright position. Block (4) consisted of only masked faces in inverted position. And Block (5) presented only faces with no masks in an inverted position. Blocks (1) and (6) were repeated 10 times each, whereas Blocks (2)–(5) were repeated 20 times each (32 × 10 + 32 × 10 + 16 × 20 + 16 × 20 + 16 × 20 + 16 × 20) for a total of 1,920 trials. Ten trials of training preceded each block.

All the experiments were run with the Macromedia Authorware software (Macromedia, 1987). On each trial, a single face was presented on the center of the screen until the participant responded according to a predefined key-assignment (“z” or “m”). Participants used the index fingers of the left and right hand, respectively. After responding, the image was removed from the screen, and the screen remained empty for 100 ms. In Experiment 1 participants were asked to categorize eight identities into two arbitrary social groups. Each group consisted of the same number of male/female and old/young identities. In Experiment 2–4 eight identities (with equal number of female/male, old/young, and angry/neutral aspects) were categorized according to emotion, age, and gender, respectively. The experiments were conducted online. In all analyses RTs smaller than 150 ms or larger than 2,800 ms were removed from analysis.

## 7. Results

Data processing was executed with R Core Team (2017). Data sets of all experiments can be downloaded from https://data.mendeley.com/datasets/96pt88tzf2/1. ANOVAs were performed with the aov function in R. Post-hot comparisons were corrected for multiple comparisons.

## 8. Experiment1: Facial Identity

### 8.1. Mixed Design

**Figure 2:**
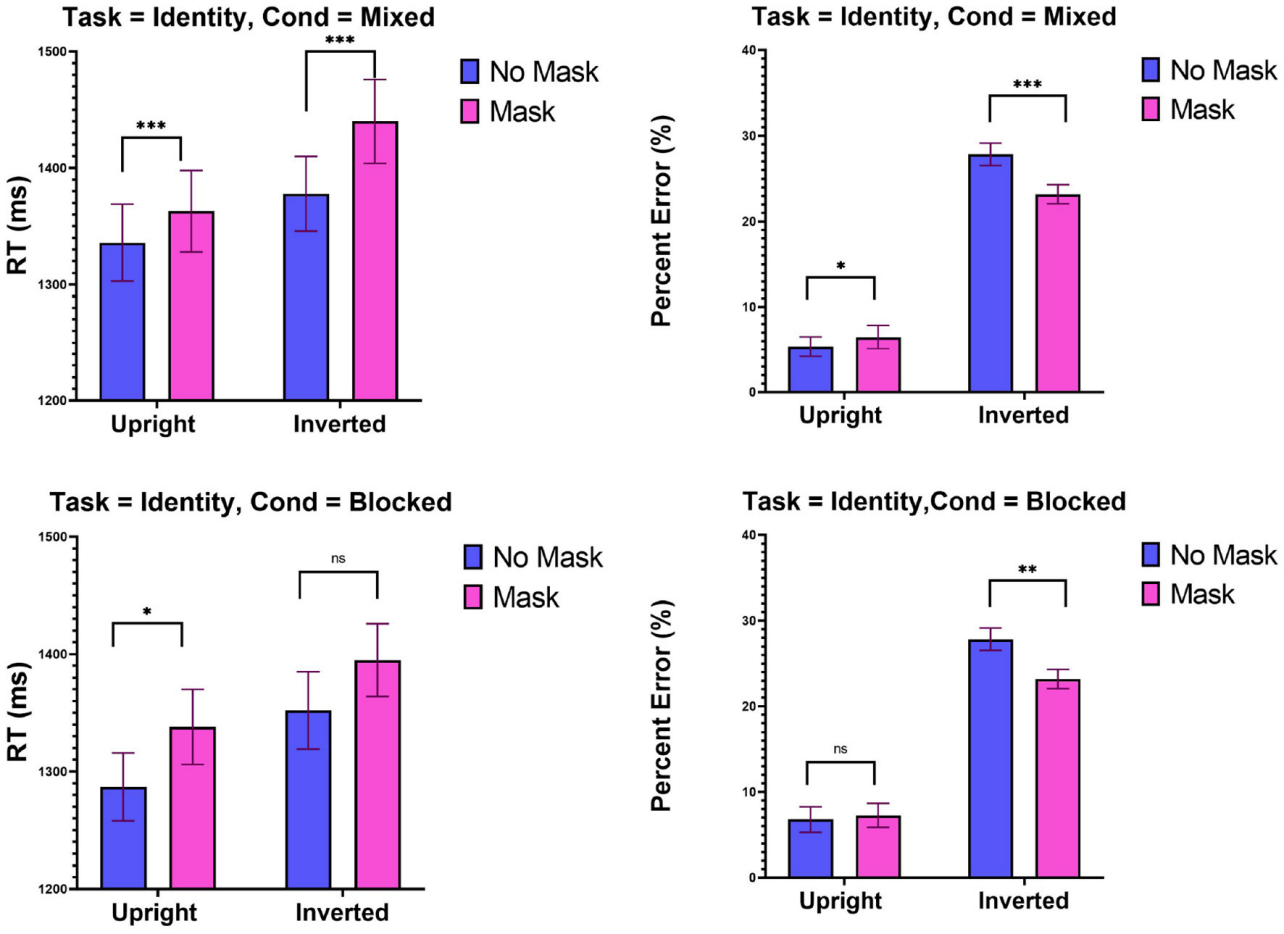
Experiment 1: Mean RTs (ms) and percent error (%) as a function of mask (mask and no mask) and inversion (upright and inverted). Top panel: Mixed condition, bottom panel blocked condition. ***p < 0.001, **p < 0.01, and *p < 0.05. Error bars are standard errors of the means.

The top-panel of Figure 2 gives means RTs and percent error in the mixed design. A Two-way ANOVA with Mask (no mask, mask) and Inversion (upright, inverted) was performed on RTs from these blocks. An effect of Mask [*F*_(1, 29)_ = 80.21, MSE = 60,519, *p* < 0.001] revealed that masked faces were identified slower than unmasked faces. A main effect of Inversion [*F*_(1, 29)_ = 4.53, MSE = 105,106, *p* < 0.005] showed that inverted faces were identified slower than upright faces. An Inversion x Mask interaction [*F*_(1, 29)_ = 6.78, MSE = 8,718, *p* < 0.05] entailed that the effect of inversion was larger in masked [*t*_(29)_ = 2.54, *p* < 0.01] compared to unmasked [*t*_(29)_ = 1.55, *p* < 0.06] condition. This result is inconsistent with the holistic account, which predicts larger inversion effects with unmasked faces. Another way of interpreting the interaction is to view it as the modulation of the effect of Mask by Inversion. The detrimental effect of mask was larger when the faces were inverted [*t*_(29)_ = 7.25, *p* < 0.0001] than when the faces were upright [*t*_(29)_ = 3.51, *p* < 0.001].

Analyses on error rates revealed a main effect of Inversion [*F*_(1, 29)_ = 176.9, *p* < 0.001], confirming the robust finding that participants are more error prone when faces are inverted than upright. A highly significant interaction Mask x Inversion [*F*_(1, 29)_ = 24.92, MSE = 0.020, *p* < 0.001] revealed that the inversion effect was larger in the unmasked [*t*_(29)_ = 15.28, *p* < 0.001] than in the masked [*t*_(29)_ = 10.26, *p* < 0.001] condition, a result that is in accordance with the holistic prediction. Another way to interpret the interaction is to view it as a modulation of the effect of Mask [*F*_(1, 29)_ = 7.189, *p* < 0.05] by inversion. In the upright condition, participants committed more errors with masked than with unmasked faces [*t*_(29)_ = 2.04, *p* < 0.05]. An opposite pattern was found with inverted faces [*t*_(29)_ = 4.48, *p* < 0.001], such that more errors were committed with non-masked than with masked faces. This finding may be well accommodated by holistic processing.

#### 8.1.1. Blocked Design

The bottom-panel of Figure 2 presents means RTs and percent error. A Two-way ANOVA with Mask (no mask, mask) and Inversion (upright, inverted) was performed on RTs from these blocks. An effect of Mask [*F*_(1, 29)_ = 4.78, MSE = 66,278, *p* < 0.05] revealed that masked faces were identified slower than unmasked faces. A main effect of Inversion [*F*_(1, 29)_ = 12.68, MSE = 11,126, *p* < 0.005] showed that inverted faces were identified slower than upright faces. A Mask x Inversion interaction was far from significance [F < 1].

Analyses on error rates revealed a highly significant effect of Inversion [*F*_(1, 29)_ = 206.00, MSE = 1.02, *p* < 0.001], such that participants committed more errors with inverted than with upright faces. A highly significant Mask x Inversion interaction [*F*_(1, 29)_ = 10.24, MSE = 0.019, *p* < 0.005] entailed that the inversion effect was larger with unmasked [*t*_(29)_ = 15.28, *p* < 0.001] than with masked [*t*_(29)_ = 10.26, *p* < 0.001] faces, an outcome that is in agreement with the holistic approach. The interaction also entailed that the effect of Mask [*F*_(1, 29)_ = 5.60, MSE = 0.01, *p* < 0.05] was modulated by inversion, such that masked faces were categorized with fewer errors than unmasked faces in the inverted faces [*t*_(29)_ = –3.52, *p* < 0.001], but that this effect was not present with upright faces [t<1]. The latter result replicates the phenomenon that we documented in RTs. It shows that masking can improve accuracy when the faces are inverted, while not hampering performance when the faces are upright.

## 9. Experiment 2: Facial Emotion

### 9.1. Mixed Design

**Figure 3:**
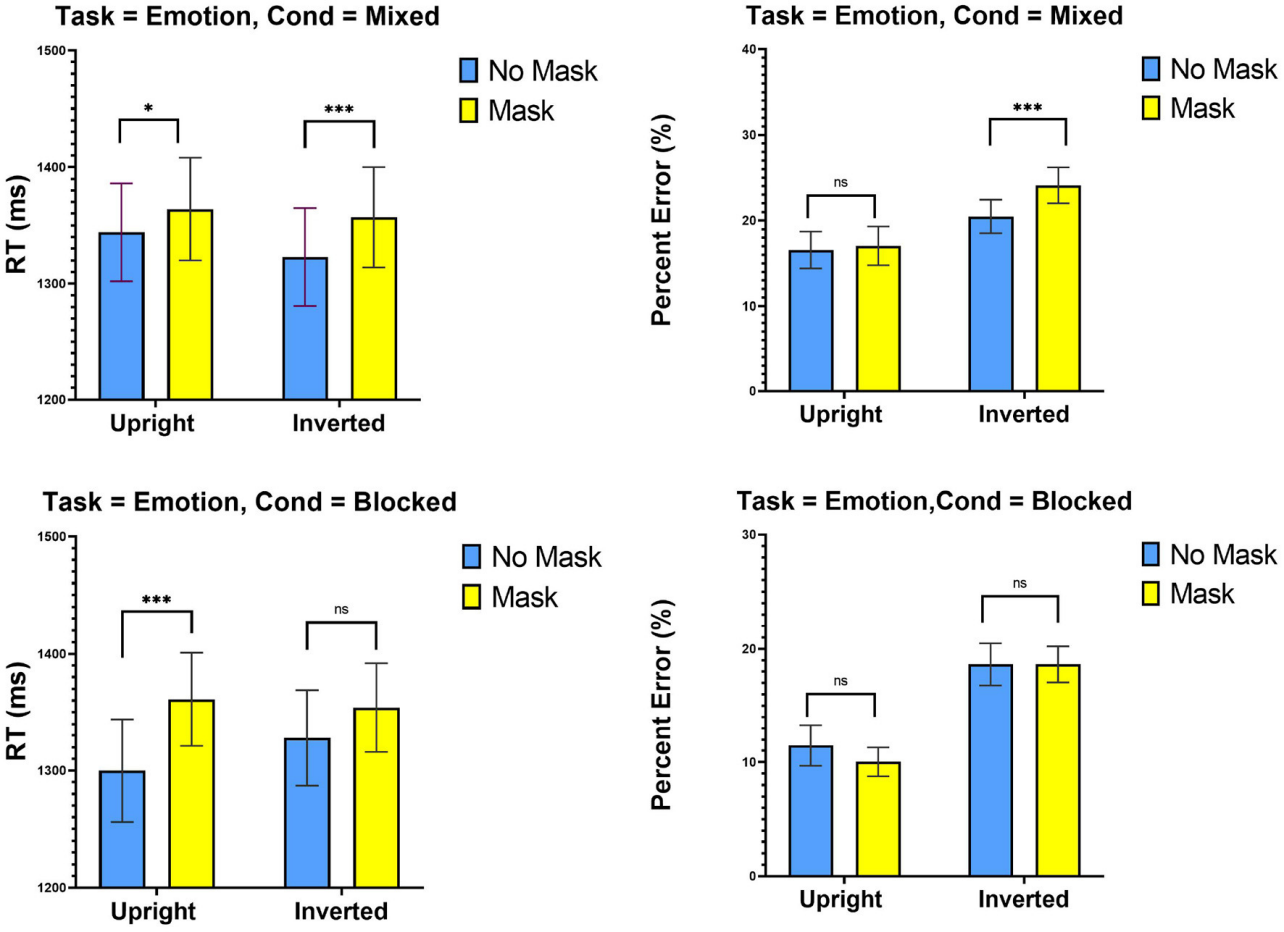
Experiment 2: Mean RTs (ms) and percent error (%) as a function of mask (mask and no mask) and inversion (upright and inverted). Top panel: Mixed condition, bottom panel blocked condition. ***p < 0.001, **p < 0.01, and *p < 0.05. Error bars are standard errors of the means.

The top-panel of Figure 3 presents means RTs and percent error in the mixed-design. A Two-way ANOVA with Mask (no mask, mask) and Inversion (upright, inverted) was performed on RTs from these blocks. The only significant effect was that of Mask [*F*_(1, 29)_ = 12.49, MSE = 21,329, *p* < 0.005], entailing slower RTs with masked than unmasked faces. The effect of Inversion [*F* < 1] and its interaction with Mask [*F*_(1, 29)_ = 1.54, MSE = 1,591, *p* = 0.22] were not significant.

Analyses on error rates revealed an effect of Mask [*F*_(1, 29)_ = 6.74, MSE = 0.005, *p* < 0.05], suggesting that, overall, participants made more errors with masked than with unmasked faces. An effect of Inversion [*F*_(1, 29)_ = 38.33, MSE = 0.185, *p* < 0.001], confirmed that participants made more errors with inverted than with upright faces. An Mask x Inversion interaction [*F*_(1, 29)_ = 3.00, MSE = 0.0035, *p* = 0.09] was not significant, an outcome that is inconsistent with the holistic account prediction.

#### 9.1.1. Blocked Design

The bottom-panel of Figure 3 presents means RTs and percent error in the blocked design. A Two-way ANOVA with Mask (no mask, mask) and Inversion (upright, inverted) was performed on RTs from these blocks. An effect of Mask [*F*_(1, 29)_ = 7.48, MSE = 56,851, *p* < 0.05] revealed slower RTs with masked compared to unmasked faces. The effect of Inversion [*F* < 1] and its interaction with Mask [*F*_(1, 29)_ = 1.41, MSE = 9,028, *p* = 0.24] were not significant. Comparable analyses on error rates revealed only effect of Inversion [*F*_(1, 29)_ = 54.74, MSE = 0.186, *p* < 0.001]. The effect of mask [*F* < 1] and its interaction with Inversion [*F*_(1, 29)_ = 1.56, MSE = 0.001, *P* = 0.22] were not significant.

## 10. Experiment 3: Facial Age

### 10.1. Mixed Design

**Figure 4:**
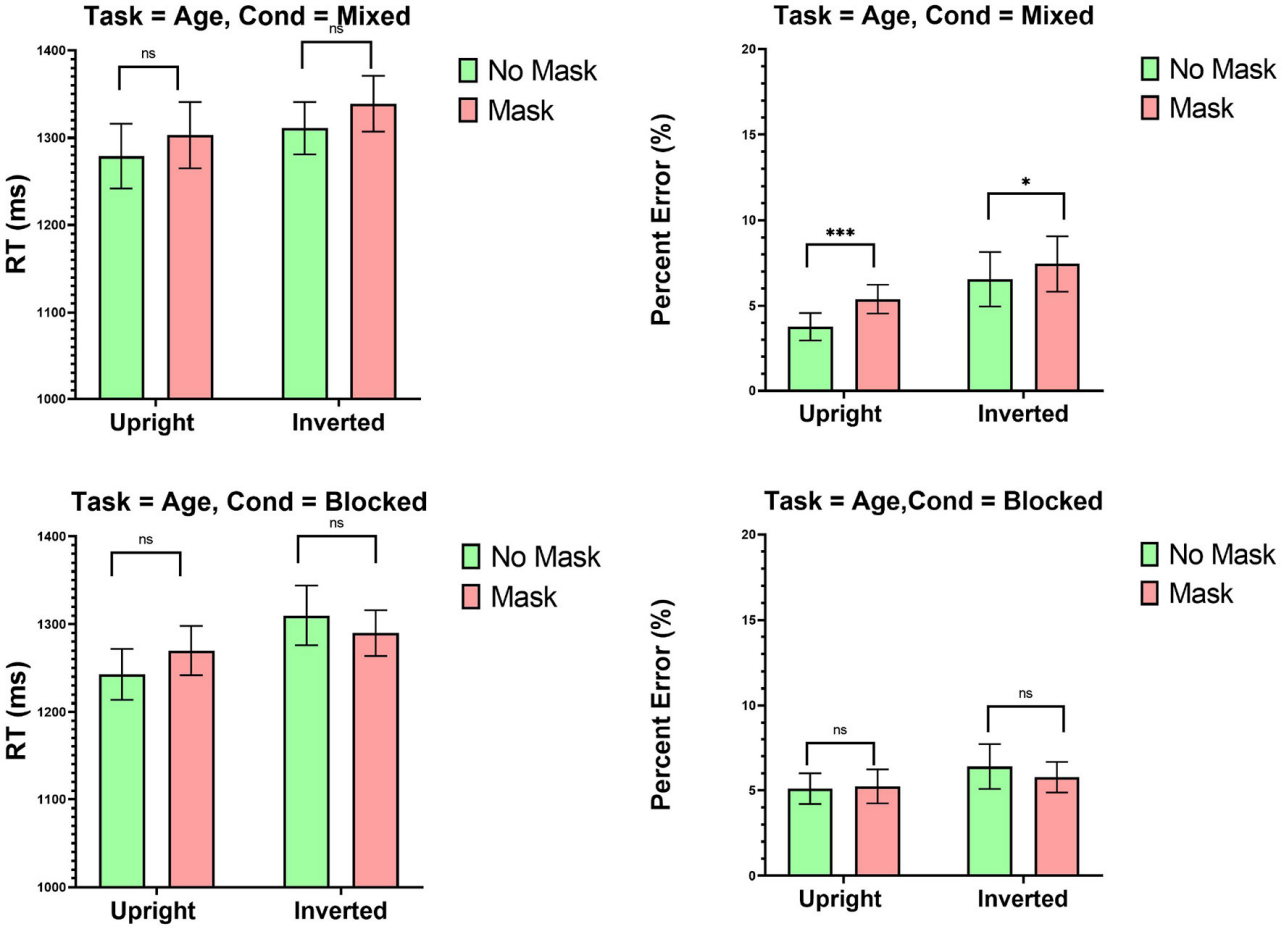
Experiment 3: Mean RTs (ms) and percent error (%) as a function of mask (mask and no mask) and inversion (upright and inverted). Top panel: Mixed condition, bottom panel blocked condition. ***p < 0.001, **p < 0.01, and *p < 0.05. Error Bars are Standard Errors of the Means.

The top-panel of Figure 4 presents means RTs and percent error in the mixed design blocks. ANOVA on RTs revealed an effect of Inversion [*F*_(1, 23)_ = 28.12, MSE = 15,961, *p* < 0.001]. The effects of Mask [*F*_(1, 23)_ = 2.95, MSE = 28,136, *p* = 0.09], and the interaction were not significant [*F* > 1]. Analyses on error revealed an effect of Inversion [*F*_(1, 23)_ = 4.93, MSE = 0.013, *p* < 0.05], such that participants committed more errors with inverted than with upright faces. Most importantly, an effect of Mask [*F*_(1, 23)_ = 17.62, MSE = 0.0041, *p* < 0.001] was observed, entailing that participants were more error prone with masked than with unmasked faces. The interaction was not significant [F<1].

#### 10.1.1. Blocked Design

The bottom-panel of Figure 4 presents means RTs and percent error in the blocked design. ANOVA on RT revealed an effect of Inversion [*F*_(1, 23)_ = 12.93, MSE = 45,441, *p* < 0.005]. The effect of Mask was far from significance [F<1]. Analyses on error revealed no particular influences of either Inversion or Mask. The effect of Inversion [*F*_(1, 23)_ = 1.35, MSE = 0.0021, *p* = 0.25], the effect of Mask [*F* < 1], and their interaction [F<1] were not significant.

## 11. Experiment 4: Facial Gender

### 11.1. Mixed Design

**Figure 5:**
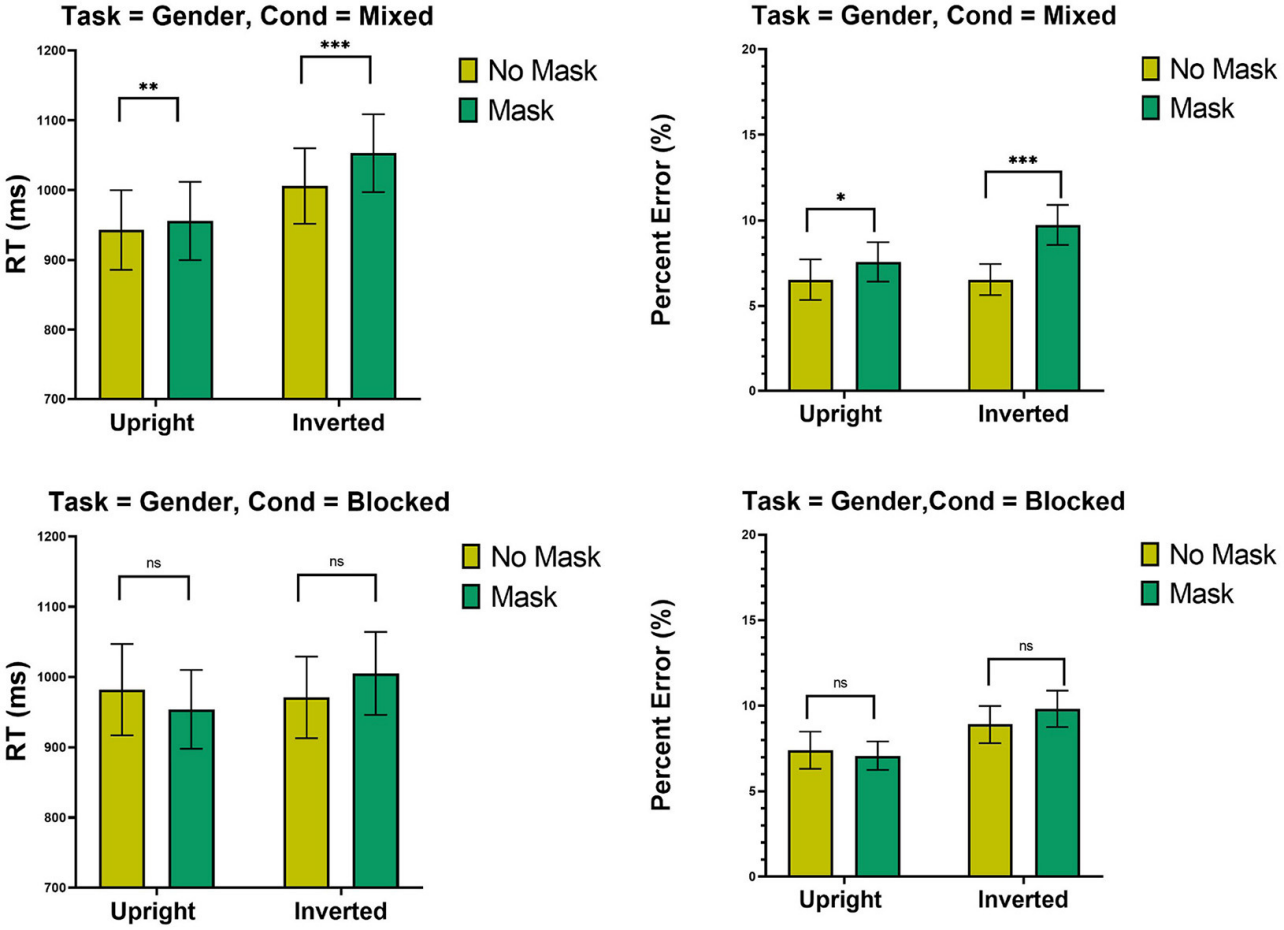
Experiment 4: Mean RTs (ms) and percent error (%) as a function of mask (mask and no mask) and inversion (upright and inverted). Top panel: Mixed condition, bottom panel blocked condition. ***p < 0.001, **p < 0.01, and *p < 0.05. Error bars are standard errors of the means.

The top-panel of Figure 5 presents means RTs and percent error in the mixed design blocks. ANOVA on RTs revealed an effect of Inversion [*F*_(1, 31)_ = 8.44, MSE = 203,766, *p* < 0.01], suggesting that inverted faces were classified slower than upright faces. The effects of Mask [*F*_(1, 31)_ = 34.71, MSE = 28,582, *p* < 0.001] and its interaction with Inversion [*F*_(1, 31)_ = 19.38, MSE = 8,776, *p* < 0.001] were significant. The latter showed that the inversion effect was larger for masked [*t*_(31)_ = 3.36, *p* < 0.001] than for unmasked [*t*_(31)_ = 2.36, *p* < 0.05] faces. This outcome is contrary to the holistic prediction. The interaction also implied that masks had larger effect on inverted [*t*_(31)_ = 5.83, *p* < 0.001] than on upright faces [*t*_(31)_ = 3.28, *p* < 0.005]. Analyses on error rates showed an effect of Mask [*F*_(1, 31)_ = 21.31, MSE = 0.014, *p* < 0.001], suggesting that participants made more errors with masked than with unmasked faces. The effect of Inversion was not significant [*F*_(1, 31)_ = 1.02, MSE = 0.003, *p* = 0.32]. An interaction [*F*_(1, 31)_ = 4.91, MSE = 0.0036, *p* < 0.05] implied larger FIE with masked [*t*_(31)_ = 1.80, *p* < 0.05] than with unmasked [*t* < 1] faces, an outcome that is contrary to the holistic predictions. The interaction also suggested that the effect of Mask was larger with inverted faces [*t*_(31)_ = 3.98, *p* < 0.001] than with upright faces [*t*_(31)_ = 2.09, *p* < 0.05].

#### 11.1.1. Blocked Design

The bottom-panel of Figure 5 presents means RTs and percent error in the blocked design. ANOVA on RT revealed no effects whatsoever (all Fs<1). Analyses on error revealed only effect of Inversion [*F*_(1, 31)_ = 7.44, MSE = 0.014, *p* < 0.05], confirming that participants made more errors with inverted than upright faces.

## 12. Discussion

Wearing face masks has become an important measure in reducing the rates of transmitting respiratory diseases (Van der Sande et al., 2008). Since the outbreak of the COVID-19 pandemic mask, it has become a regular practice in many countries over the world. This practice exerts tremendous impact on many aspects of our social life. The current study provided a systematic evaluation of the impact of face masks on the perception of primary face dimensions (identity, emotion, age, and gender). Four experiments were conducted in which different groups of participants engaged in a speeded categorization task with masked and unmasked faces. In each experiment, a different group of participants classified the same set of faces according to a predefined facial dimension (identity, emotion, age, and gender). This allowed us to measure the impact of face masks in terms of both speed and accuracy. We also tested the influences of inversion and processing strategies (using blocked and mixed designs) on face categorization.

The results of the four experiments are summarized in Table 1. As can be noted, masks affected performance for virtually all tested facial dimensions. However, there was considerable variance in this influence as a function of the dependent variable (speed vs. accuracy) and type of context (mixed vs. blocked design). First, with respect to identity judgments, we found that masks indeed reduced both speed and accuracy for the categorization of upright faces, with the exception of accuracy in the blocked condition. The prediction that the FIE should be reduced for masked faces (Freud et al., 2020) was not fully corroborated. It was supported in the accuracy data, but not in the speed data. Overall, masks exerted stronger influences in the mixed compared to the blocked design. This may suggest that observers have adopted processing strategies to mitigate some of the detrimental consequences of masks in that context. We also found an interesting pattern by which masks improved accuracy (but not speed) when faces were inverted. This may suggest that when the task becomes more demanding, people change the way they process faces, and start to focus on the more distinguishing facial details.

**Table 1:** Summary: Effects of masks on categorization of faces in the four experiments.

**Experiment**	**Dimension**	**Condition**	**Effect of mask**			
			**Upright**		**Inverted**	
			**RT**	**Error**	**RT**	**Error**
1	Identity	Mixed	v	v	v	v
		Blocked	v			v
2	Emotion	Mixed	v		v	v
		Blocked	v			
3	Age	Mixed		v		v
		Blocked				
4	Gender	Mixed	v	v	v	v
		Blocked				

With respect to emotion categorization, masks impaired both speed and accuracy in the mixed design, and speed in the blocked design. It should be noted that the emotions in our study were limited to anger and neutral expressions. The results, however, are compatible with previous studies (Kret and De Gelder, 2012; Carbon, 2020; Grundmann et al., 2021) who found disruption of emotion recognition due to face masks. Our findings have several implications. First, measures of accuracy and speed can be dissociated. Second, the influence of type of design (blocked vs. mixed) entails that observers may have acquired various processing strategies that allowed them to mitigate at least part of the unwarranted influences of masks on their perception of facial emotion. Third, contrary to the holistic prediction, when the FIE was observed (in the blocked design) it did not appear to be smaller with masked faces. The general impedance of emotion recognition is consistent with a recent study by Carbon (2020) who presented observers with emotional faces with or without masks. He found that accuracy was hampered for most of the canonical emotional expressions (excepting fearful and neutral faces). A disruption of emotion recognition has been documented with other types of occlusions, such as covering the mouth area with cardbord (Bassili, 1979), the Bubbles technique (Blais et al., 2012), or a shawl or a cap (Kret and De Gelder, 2012). With respect to facial age, masks had decreased only the accuracy of age categorization in the mixed block for upright and inverted faces. Importantly, under this condition, the FIE was not modulated by the effect of mask. These outcomes suggests that: (a) participants adopt different strategies for extracting facial age, and (b) extraction of the age dimension from faces might not be sustained by holistic processing. Finally, with respect to gender, masks impaired both speed and accuracy of upright and inverted faces, but only in the mixed block design. In contrast to the holistic prediction, the FIE was larger with masked than with unmasked faces. This entails that: (a) gender categorization can be impaired by masks, (b) this impairment is not accounted by holistic processing, and (c) participants adopt different strategies to deal with the impact of masks when extracting facial age. In sum, we did not find evidence for impairment of holistic processing as far as the FIE is indeed a marker of holism (see for, Sekuler et al., 2004). We did find evidence for the notion that masks exert their influence in a selective manner, interfering with some of aspects of performance but not with others, depending on context.

Social interactions rely on fast and correct perception of facial identity, emotion, age, and gender. Wearing masks may complicate social interactions because masks interfere with the extraction of these basic dimensions. This of course should not lead people to refrain from wearing masks. There are medical situations in which masks can be instrumental in preventing the propagation of a disease and consequently serious illness and death. On the more optimistic side, we note that people can learn how to mitigate at least part of the unwarranted effects of masks. This was evident in reduction of the harmful effects of masks in the blocked designs in the current experiments. We subsume that people can be adaptive and compensate for distortion and scarcity of information caused by face masks. To optimize their performance, observers can employ various cognitive strategies. For example, observers can engage in an efficient cue-integration from various sources and modalities (Massaro and Palmer, 1998). In this sense, inferences regarding primary facial dimensions such as identity, emotion, gender or age, can be made based on other cues and modalities than the obvious ones. These include: voice (Golan and Baron-Cohen, 2006; Knoblauch et al., 2021), gate (Cutting and Kozlowski, 1977), body gestures (Aviezer et al., 2012), and other social context of a situation (Mondloch, 2012). For example, voice characteristics can assist observers to disambiguate gender (Knoblauch et al., 2021).

## 13. Implications and Future Directions

Faces are the most important means by which we transmit and decode information about ourselves and others. The need to wear face masks during social interaction poses considerable challenges to normal communication. This has changed interpersonal interactions in many ways, likely permanently. Face masks not only hamper actual information transmission, they also create negative feelings of alienation and emotional distancing (Grundmann et al., 2021). For example, the quality of tutoring in schools significantly diminishes when teacher's and pupils' faces are occluded by masks (Nobrega et al., 2020); patients may feel less compassion and care when their caregivers are wearing masks (Marler and Ditton, 2021); people with hearing loss may not benefit anymore from lip-reading (Chodosh et al., 2020).

To overcome, at least part, of these devastating consequences, several measures can be taken. For example, our study revealed that people can improve their ability to categorize masked faces if they operate in an environment in which all people are wearing masks in comparison to a mixed environment in which only some of the faces are occluded. One explanation to this finding is that in such an environment, people do not pay the cost of switching between processing strategies. Moreover, our results indicate that the detrimental effects of masks are not related to a holistic strategy of processing. This entails that the reduction in speed and accuracy, when observed, is probably due to disruption of part-based processing. One implication of this finding is that people can learn to concentrate on small details in faces that are informative, and in this way compensate for the lost features. For example, people in our study exhibited an improved accuracy for masked faces (even in comparison to unmasked faces) when faces were presented upside down. This may imply that teaching people how to attend to distinguishing facial features can improve their ability to perceive basic aspects.

A creative solution to mitigate social disconnection resulting from facial feature occlusion is the personal protective equipment (PPE) project (Molnar-Szakacs et al., 2021). This project started during the 2014–2015 Ebola outbreak and was revised in Stanford. The basic idea behind this project is to add to medical staff's PPE gown a photo sticker with their image. This allows the patient to perceive the identity, gender, and age of the caregiver, and therefore better relate to them. This and similar projects can be helpful in overcoming the detrimental effects on accuracy and speed of processing of primary face dimensions documented in this study. Another solution is that of using transparent masks in which the often occluded parts of the face can been seen. A recent study (Yi et al., 2021) finds that transparent masks have positive effects on speech intelligibility. These masks can help people with hearing loss to benefit from lip-reading, and likely to facilitate more efficient recognition of primary facial dimensions.

## 14. Limitations and Advantages

The current study enjoys a high level of ecological validity. Our faces conveyed real people who were photographed with and without real masks. This in contrast to the great majority of previous studies who have artificially patched a generic mask on images of unmasked faces. This practice is dubious, because real masks can convey valuable structural information via curvature, depth, light-shading, and other types of cues. However, one limitation of the current study is that observers saw the same images repeatedly and therefore could use part-based clues, such as the relative size of the mask to the face, to identify a person. Similar visual clues may explain the weak evidence for holistic processing. While masks disrupt the face gestalt, they could also provide additional distinctive cues if the mask-on picture is always the same. Conversely, this is the reason why “in the wild” people are buying peculiar and unique masks, to aid recognition and affirm individuality.

## 15. Conclusions

The current results reveal that the COVID-19 masks pose a real challenge to our everyday social interaction. We have shown that masks hinder major aspects of social perception, as they interfere with normal speed and accuracy of extracting identity, emotion, age, and gender. We have demonstrated though, that the influences of masks are not due to the breakdown of holistic processes. Moreover, we found that the impact of masks is not automatic and that under some contexts observers can control at least part of the unwarranted effects. Our investigation also reveals a dissociation between accuracy and speed. This entails that a more complete model such as the diffusion model (Ratcliff, 1978; Fitousi, 2020c) should be applied in future studies to explain the interplay between speed and accuracy. Finally, we have shown that the FIE is generally not modulated by masks in the direction predicted by the holistic account (Farah et al., 1998; Freud et al., 2020). If anything, our results are consistent with the view that masks hinder specific features that are relevant for analytic perception (Fitousi, 2015, 2021), which is highly diagnostic for social categorization.

## Data Availability Statement

The datasets presented in this study can be found in online repositories. The names of the repository/repositories and accession number(s) can be found in the article/supplementary material.

## Ethics Statement

The studies involving human participants were reviewed and approved by the study was approved by Ariel University Ethical Committee (AU-SOC-DF-20210411). The patients/participants provided their written informed consent to participate in this study. Written informed consent was obtained from the individual(s) for the publication of any potentially identifiable images or data included in this article.

## Author Contributions

DF conceived and designed the study and analyzed the data and wrote the manuscript. CP and NR created the face images and run the Experiments. OA run the experiments. All authors contributed to the article and approved the submitted version.

## Funding

This research was supported by the Israel Science Foundation (grant No. 1498/21).

## Conflict of Interest

The authors declare that the research was conducted in the absence of any commercial or financial relationships that could be construed as a potential conflict of interest.

## Publisher's Note

All claims expressed in this article are solely those of the authors and do not necessarily represent those of their affiliated organizations, or those of the publisher, the editors and the reviewers. Any product that may be evaluated in this article, or claim that may be made by its manufacturer, is not guaranteed or endorsed by the publisher.
